# Effect of Point Mutations on Structural and Allergenic Properties of the Lentil Allergen Len c 3

**DOI:** 10.3390/membranes11120939

**Published:** 2021-11-27

**Authors:** Daria N. Melnikova, Ekaterina I. Finkina, Ivan V. Bogdanov, Anastasia A. Ignatova, Natalia S. Matveevskaya, Andrey A. Tagaev, Tatiana V. Ovchinnikova

**Affiliations:** 1 M.M. Shemyakin & Yu.A. Ovchinnikov Institute of Bioorganic Chemistry, The Russian Academy of Sciences, Miklukho-Maklaya Str., 16/10, 117997 Moscow, Russia; finkina@mail.ru (E.I.F.); contraton@mail.ru (I.V.B.); aignatova_83@mail.ru (A.A.I.); andazitag@yandex.ru (A.A.T.); ovch@ibch.ru (T.V.O.); 2 Phystech School of Biological and Medical Physics, Moscow Institute of Physics and Technology (State University), 141701 Dolgoprudny, Russia; 3 G.N. Gabrichevsky Research Institute for Epidemiology and Microbiology, Admiral Makarov St., 10, 125212 Moscow, Russia; matveevskaya@mail.ru

**Keywords:** Len c 3, site-directed mutagenesis, recombinant analogue, allergen, IgE-binding capacity, lipid binding, gastroduodenal digestion

## Abstract

Plant lipid transfer proteins (LTPs) are known to be clinically significant allergens capable of binding various lipid ligands. Recent data showed that lipid ligands affected the allergenic properties of plant LTPs. In this work, we checked the assumption that specific amino acid residues in the Len c 3 structure can play a key role both in the interaction with lipid ligands and IgE-binding capacity of the allergen. The recombinant analogues of Len c 3 with the single or double substitutions of Thr41, Arg45 and/or Tyr80 were obtained by site-directed mutagenesis. All these amino acid residues are located near the “bottom” entrance to the hydrophobic cavity of Len c 3 and are likely included in the IgE-binding epitope of the allergen. Using a bioinformatic approach, circular dichroism and fluorescence spectroscopies, ELISA, and experiments mimicking the allergen Len c 3 gastroduodenal digestion we showed that the substitution of all the three amino acid residues significantly affected structural organization of this region and led both to a change of the ligand-binding capacity and the allergenic potential of Len c 3.

## 1. Introduction

The spread of food allergies in developed countries has become one of the most serious health issues. Today, about 30% of the world population suffers from allergic-related diseases [1,2]. IgE-mediated allergic reaction is one of the most common and severe types of allergic reactions to foodstuffs, and it is characterized by elevated allergen-specific IgE levels in blood of atopic patients [3]. The IgE-mediated food-allergic reactions result in skin (urticaria, angioedema), respiratory tract (rhinitis, asthma), gastrointestinal tract (abdominal pain, diarrhea), and systemic anaphylactic reactions [4,5].

Legumes are a staple food in the diet of many countries due to their high protein content and low price. Widespread consumption of legumes has led to an increase in the prevalence of legume allergy. Additionally, there is a high rate of cross-reactivity between different legume allergens. Most previous studies have been focused on 2 members of the legume food family, peanuts and soybeans, but lentils should also be examined in detail as a basic food in the Mediterranean area, Near East, India, Pakistan and the United States [6]. It has been shown that allergy to lentil is the most frequently diagnosed legume allergy in children from a Mediterranean area [7]. Allergy to lentils started early in life and is characterized by predominantly mild allergic symptoms, like oral allergy syndrome and urticaria; however, in some cases, lentils are able to cause severe systemic reactions like anaphylaxis [8,9].

Previously, a new allergen Len c 3 belonging to the class of lipid transfer proteins (LTPs) was isolated from the lentil *Lens culinaris* seeds [10]. We have shown that Len c 3 was cross-reacting with the major peach allergen Pru p 3 [10]. In Len c 3, four conserved disulfide bridges stabilize a compact spatial structure containing four helices and a central hydrophobic cavity [11]. The protein is able to bind with various effectiveness of such ligands as fatty acids (FAs) and lysolipids in solutions with different pHs [11,12]. This allergen is sensitive to heating and duodenal digestion, but the presence of its diverse ligands may increase the protein thermostability, decrease the rate of its gastroduodenal degradation, but not affect the IgE-binding capacity of the allergen [13].

Site-directed mutagenesis can be used to identify key amino acid residues, either responsible for the ability of LTPs to bind different lipid ligands [14], or playing an important role in the IgE-binding capacity of the proteins [15]. In our previous work, we assumed that the same amino acid residues can participate both in the interaction with lipid ligands and specific IgE from the sera of allergic patients [16]. Therefore, the main goal of this study was to investigate the effect of point amino acid substitutions on the structural and allergenic properties of Len c 3. With this aim in view, we obtained and characterized recombinant analogues of the allergen with single or double substitutions of Thr41, Arg45 and Tyr80, which are located close to each other near the “bottom” entrance to the hydrophobic cavity of Len c 3 and are apparently included in the IgE-binding epitope of the allergen.

## 2. Materials and Methods

### 2.1. Materials

2-p-toluidinonaphthalene-6-sulphonate (TNS) was purchased from Sigma-Aldrich (St. Louis, MO, USA). Digestive enzymes (porcine pepsin and trypsin, bovine α-chymotrypsin) were purchased from Sigma-Aldrich (St. Louis, MO, USA).

Len c 3 and its R45A, Y80A and R45A/Y80A analogues were produced as earlier described [11]. The T41A and T41A/Y80A analogues were obtained by site-directed mutagenesis of the original plasmid pET-His8-TrxL-Len c 3 using full-length inverse PCR amplification with mutagenizing primers (Supplementary Materials, Table S1). All recombinant proteins were overexpressed in *Escherichia coli* and purified as described previously [11]. Homogeneity and identity of the recombinant protein samples were confirmed by MALDI mass spectrometry (Supplementary Materials, Figure S1). Rabbit polyclonal anti-Len c 3 antibodies were obtained as previously described [17].

Sera from patients (*n* = 100) with legume and other plant food allergy were obtained from the Clinical Diagnostic Center of the G.N. Gabrichevsky Research Institute for Epidemiology and Microbiology. The work with human sera was approved by the local ethical committees of the Institute (FS-99-01-009026, Moscow, Russia). The amount of specific IgE to allergen extracts in the patient sera was determined by using RIDA qLine Allergy Panel 1–4 (R-Biopharm, Pfungstadt, Germany). Screening of sera containing specific IgE (sIgE) to Len c 3 was performed by Enzyme-Linked Immunosorbent Assay (ELISA). Sera samples from non-allergic individuals were used as a negative control. Sera of 14 allergic patients containing sIgE to Len c 3 were selected for experiments on IgE-binding with the recombinant analogues carrying amino acid substitutions. Seven serum samples were used in experiments with the T41A, Y80A and T41A/Y80A analogues, and the others were used in experiments with the R45A, Y80A and R45A/Y80A analogues.

### 2.2. Circular Dichroism Spectroscopy

Circular dichroism spectra were recorded at 25 °C using a J-810 spectropolarimeter (Jasco) in a 0.1 cm path length quartz cell (Hellma GmbH & Co. KG, Mullheim, Germany) in a wavelength range of 190–250 nm (scan rate 1 nm) using solutions of the recombinant proteins in 10 mM phosphate buffer, pH 7.4, at a concentration of 17 mM.

### 2.3. Ligand Binding

An ability of Len c 3 and its mutant analogues (T41A, R45A, Y80A, T41A/Y80A, R45A/Y80A) to bind lipids was assessed using the TNS fluorescent probe as previously described [18]. Fluorescence intensity was measured at 437 nm with excitation at 320 nm using F-2710 spectrofluorometer (Hitachi High Technologies America Inc., Pleasanton, CA, USA). TNS (4 µM) with or without a lipid (4 µM) was incubated for 1 min in a stirred cuvette containing 2 mL of the 10 mM phosphate buffer (pH 7.4) with gentle mixing before the initial fluorescence (F_0_) was recorded. Then, Len c 3 or its mutant analogues (4 µM) were added, and 2 min later the fluorescence was recorded at equilibrium (F). Experiments were performed in triplicate. The results were expressed as a percentage of the allergen-TNS complex fluorescence calculated according to the formula [(F − F_0_)/F_C_] × 100%, where F_C_ is the fluorescence of the allergen-TNS complex in the absence of a lipid.

### 2.4. Gastroduodenal Digestion In Vitro

Gastroduodenal digestion of the recombinant Len c 3 and its mutant analogues in vitro was simulated as described with some modifications [13]. Cleavage mimicking the protein gastric digestion in vitro was performed for 2 h at 37 °C using 50 ng (0.1 U) of pepsin per 1 μg of Len c 3 or its mutant analogues in 0.05 M HCl, pH 2.0 (at the final protein concentration of 0.05 mM). For cleavage mimicking subsequent protein duodenal digestion in vitro, pH of the mixture resulting from gastric digestion was adjusted to 8.0 by addition of 0.1 M ammonium bicarbonate and incubated for 24 h at 37 °C with 2.5 ng (0.03 U) of trypsin and 10 ng (0.4 × 10^−3^ U) of α-chymotrypsin per 1 μg of the substrate (Len c 3 or its mutant analogues). Digestion of all the proteins was monitored by sodium dodecyl sulfate polyacrylamide gel electrophoresis (SDS-PAGE) [19]. SDS-PAGE was carried out in Tris-Glycine electrode buffer, pH 8.3, using disk-gels with 3% stacking and 15% separating gels. All samples were mixed with loading buffer containing 2-mercaptoethanol besides SDS. Each experiment was carried out three times. Analysis of the obtained polyacrylamide gels was performed using Gel Doc XR^+^ imaging system (Bio-Rad, Hercules, CA, USA) and Image Lab Software.

### 2.5. Bioinformatic Approaches to Study Structural Properties of Mutant Analogues of Len c 3

Spatial structure of the wild-type lentil Len c 3 has been solved by us previously [PDB ID 2MAL] using heteronuclear NMR in solution [11]. This structure was used in the current study as a template. The mutant analogues T41A, R45A, Y80A, T41A/Y80A, and R45A/Y80A were generated by means of the mutagenesis tool of the PyMOL 1.8.2.0 software (Schrödinger, LLC, New York, NY, USA) [20]. The hydrophobic pockets’ volumes were calculated using the MOLE online tool (https://mole.upol.cz/, accession date 21 October 2021) with a 5 Å probe radius [21]. Proteins were visualized by the PyMOL 1.8.2.0 software, and hydrophobicity coloring was made by using *color_h* script, which was based on a normalized consensus Eisenberg scale [22].

### 2.6. Immunoglobulin Binding Assay

Comparative IgE-binding assay was performed by ELISA using sera from allergic patients containing specific IgE to Len c 3. For that, plate wells (Corning Incorporated, Corning, NY, USA) were coated with the recombinant Len c 3 and its mutant analogues (0.5 mg/well) in 0.01 M phosphate-buffered saline (PBS), pH 7.4, for 1 h, at 37 °C; saturated with 2% bovine serum albumin (BSA, SERVA, Heidelberg, Germany) in PBS buffer for 1 h, at 37 °C; and then incubated with sera of allergic patients (a dilution ratio of 1:5) overnight, at 4 °C. sIgE-binding was detected by using the peroxidase-conjugated anti-human IgE from goat (a dilution ratio of 1:2000, Sigma) in PBS with 0.5% BSA, and 3,3′,5,5′-tetramethylbenzidine (TMB) liquid substrate system for ELISA (Sigma). The enzymatic reaction was stopped after 30 min by 2 N H_2_SO_4,_ and absorbance values were determined at 450 nm. PBS, containing 0.05% Tween-20 (PBS-T), was used as a washing solution on each step. Each experiment was carried out twice.

ELISA assays with the polyclonal anti-Len c 3 rabbit antiserum were performed as described above with some modifications. After blocking free binding sites, the plate wells were incubated with the anti-Len c 3 rabbit antiserum in PBS (from 1:400 to 1:735,306 serial dilutions) for 1 h at 37 °C. The peroxidase-conjugated anti-rabbit antibodies from goat (a dilution ratio of 1:50,000, Sigma) in PBS with 0.5% BSA were used for detection. Each experiment was carried out three times.

## 3. Results

### 3.1. Secondary Structure

**Figure 1:**
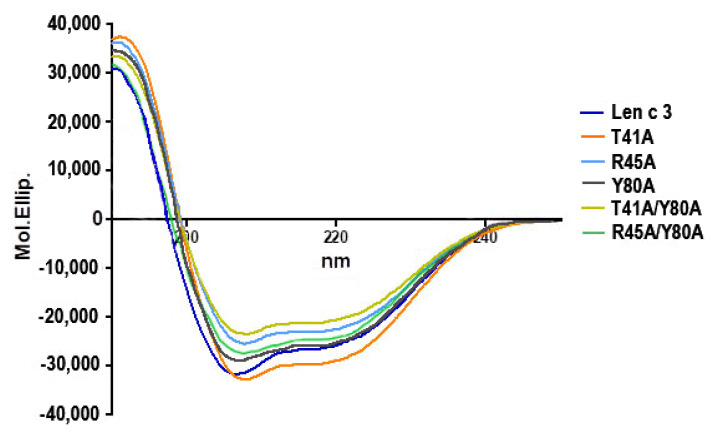
Overlay of the CD spectra of Len c 3 and its mutant analogues (T41A, R45A, Y80A, T41A/Y80A, R45A/Y80A), measured at 25 °C.

An effect of all amino acid substitutions on the secondary structure of Len c 3 was analyzed by CD spectroscopy (Figure 1). The far-UV CD spectra of Len c 3 and its mutant analogues (T41A, R45A, Y80A, T41A/Y80A, R45A/Y80A) at pH 7.0 showed a maximum at 192 nm and double minima at 208 and 222 nm, typical of a predominantly α-helical structure. It is worth noting that amino acid substitutions slightly increased α-helical content for all mutant analogues (Supplementary Materials, Table S2).

### 3.2. Ligand Binding Assay

**Figure 2:**
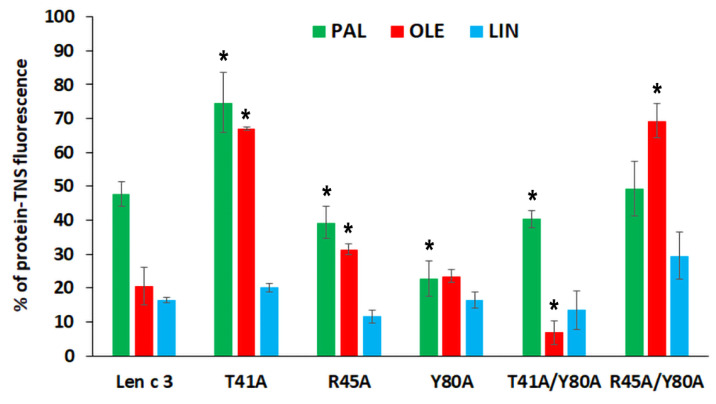
Effect of fatty acids on the fluorescence level of the protein-TNS complexes. The results are expressed as the mean values (±SD) of the percentage of the fluorescence using the protein-TNS complexes without ligands as controls. Differences in mean values between the percentage of the fluorescence of Len c 3 and its mutant analogues were estimated by Student’s *t*-test (significance level is: * *p* < 0.01).

To investigate whether Len c 3 and its mutant analogues bind to fatty acids (FAs) in aqueous solution, TNS displacement assay was used (Figure 2). TNS fluorescence is very weak in water and increases when it is dissolved in a hydrophobic medium or a fluorophore is bound to a protein. Binding of TNS to the protein hydrophobic cavity resulted in an increase in fluorescence intensity, the value of which was taken as 100% for each protein. Palmitic (PAL), oleic (OLE) and linoleic (LIN) acids having acyl chain of different lengths and degree of saturation, were used in the experiments. These FAs are present in the greatest amount in the lipid composition of lentil [23], and may be possible endogenous ligands of Len c 3.

Len c 3 and its R45A, T41A/Y80A analogues bound to all the tested FAs with an equal efficiency. In the case of Y80A, it bound PAL better than Len c 3 (22% of the control fluorescence). Surprisingly, low affinity of T41A for PAL and OLE was shown (74% and 67% of the control fluorescence, respectively). A more interesting result was obtained for R45A/Y80A. This analogue associated with OLE with low affinity (69% of the control fluorescence); however, binding capacities of PAL and LIN were similar to that of Len c 3.

### 3.3. Bioinformatic Approaches to Study Protein–Ligand Interactions

**Figure 3:**
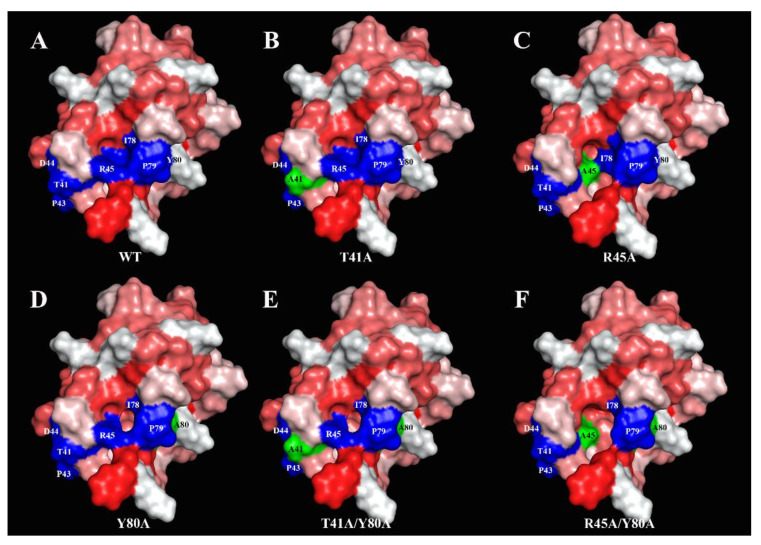
The NMR solution structure of the ligand-free Len c 3 (2MAL) (**A**) and structures of the mutant analogues of Len c 3 predicted by computer simulation (**B**–**F**). Hydrophobicity is displayed in a normalized consensus Eisenberg scale (the more positive the value, the more hydrophobic the amino acid residues). Amino acid residues crucial for IgE-binding (Thr41, Pro43, Asp44, Arg45, Ile78, Pro79, and Tyr80) [15,24] are shown in dark blue. Amino acid substitutions are displayed in green.

In order to determine the effects of amino acid substitutions on the protein structures, we calculated the changes in internal hydrophobic pockets’ volumes. According to the calculations with the use of the MOLE online tool with a 5 Å probe radius, both Len c 3 and T41A analogue hold an internal hydrophobic cavity of ∼2462 Å^3^, so it seemed that T41A substitution did not affect the cavity volume. The R45A analogue has a similar cavity volume of 2446 Å^3^. However, Y80A substitution led to the significant increase of the internal hydrophobic pocket up to 2722 Å^3^ (~10%) in the case of both Y80A and T41A/Y80A. Double substitution R45A/Y80A increased the volume of the internal hydrophobic cavity up to ∼2754 Å^3^. At the same time, the overall protein surface volume did not change significantly and varied from 6563 to 6703 Å^3^. Substitutions also increased the size and affected the shape of the “bottom” entrance to the hydrophobic cavity of Len c 3, especially in the case of double-mutant R45A/Y80A analogue (Figure 3F). Mapping of the main conformational IgE-binding epitope of the peach Pru p 3 allergen responsible for cross-reactivity between antibodies to Pru p 3 and other allergenic food LTPs [15,24] on the Len c 3 structure revealed that this epitope was located also mainly near the “bottom” entrance (Figure 3A–F, shown in dark blue). These substitutions disrupt continuity of this region of Len c 3, which is probably engaged in the interaction with specific IgE, and this disruption is especially pronounced in the case of T41A and double-mutant analogues T41A/Y80A and R45A/Y80A (Figure 3).

### 3.4. Proteolytic Cleavage Mimicking Gastroduodenal Digestion In Vitro

**Figure 4:**
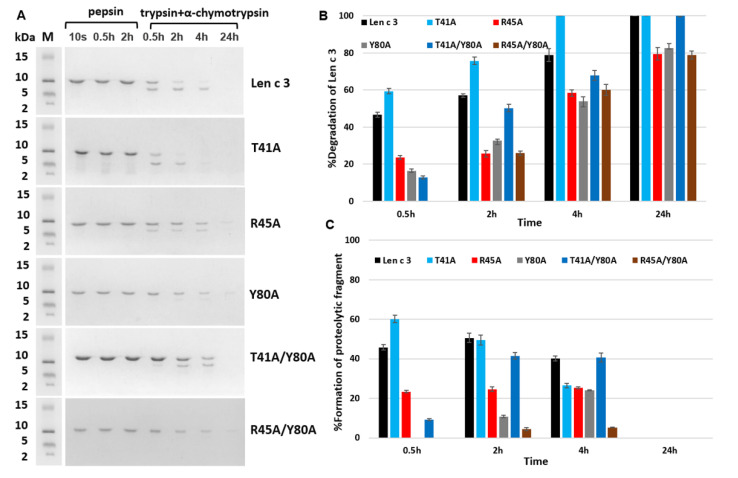
SDS-PAGE analysis of proteolytic fragments of Len c 3 and its mutant analogues after cleavage mimicking gastroduodenal digestion in vitro (**A**). M—molecular mass standards; 10 s, 0.5 h, 2 h—the proteins fragmentation by pepsin under conditions mimicking gastric digestion; 0.5 h, 2 h, 4 h, 24 h—digests after the subsequent proteins incubation with the mixture of trypsin and α-chymotrypsin mimicking their cleavage in human gut. Comparative analysis of duodenal digestion of Len c 3 and its mutant analogues (**B**,**C**). The bands corresponding to intact proteins (**B**) and their proteolytic fragments with molecular mass of 6–8 kDa (**C**) were registered using Gel Doc XR^+^ imaging system (Bio-Rad) and Image Lab Software. Percentage of degraded proteins was calculated as (So − Sa)/So × 100%, where So is an area of the protein band at the initial time (10 s) and Sa is an area of the protein band at a specified time. Error bars represent standard deviation between technical replications. Differences in percentages of Len c 3 and its mutant analogues degradation were compared by *t*-test (significance level is *p* < 0.05 for all variants). Percentage of proteolytic fragments was calculated as Sp/So × 100%, where Sp is an area of the proteolytic product band at a specified time. Analysis of gastric protein digestion is not given since Len c 3 and its mutant analogues are practically not cleaved by pepsin.

Earlier, we showed that the lentil Len c 3 allergen is not sensitive to pepsin degradation, but is quickly digested by a mixture of duodenal enzymes trypsin and α-chymotrypsin [13]. We also suggested that Tyr80 was one of the key amino acid residues at which the lentil allergen was cleaved during simulated gastroduodenal digestion with the formation of the large fragment 1–80 [13]. In order to check this assumption and also to clarify the role of Thr41 and Arg45, a comparative study of susceptibility of Len c 3 and its mutant analogues to proteolytic cleavage was carried out (Figure 4A–C).

Surprisingly, the replacement of Thr41 by alanine increased a rate of the protein degradation and the band corresponding to the mutant analogue T41A disappeared after 4 h of incubation with the mixture of trypsin and α-chymotrypsin, as shown by SDS-PAGE (Figure 4A). The replacement of Arg45 as well as Tyr80 by alanine decreased a rate of the protein digestion, and approximately 20% of the intact proteins were present in hydrolysates even after 24 h of incubation with duodenal enzymes (Figure 4A,B). Formation of the large fragment with molecular mass of approximately 8 kDa was less effective in the case of the Y80A analogue during the first 2 h of simulated duodenal digestion (Figure 4A,C). Decreased proteolysis of the R45A and Y80A analogues demonstrated that these two amino acid residues are exposed to duodenal enzymes in the allergen Len c 3 structure. Cleavage of the double-mutant analogue T41A/Y80A was slower than that of Len c 3 but faster than digestion of Y80A (Figure 4A–C). The rate of cleavage of the double-mutant analogue R45A/Y80A was not the lowest among all the studied proteins as expected and approximately corresponded to that of R45A and Y80A.

### 3.5. Immunological Properties of Len c 3 and Its Mutant Analogues

Previously, we showed that the lentil allergen Len c 3 revealed a cross-reactivity with antibodies to the peach Pru p 3 allergen, the major sensitizer of the LTP class [10]. We suggested that Len c 3 has linear and conformation epitopes similar to those of Pru p 3, which are well studied [15,24]. In this work, we investigated a possible role of three amino acid residues—Thr41, Arg45 and Tyr80, presumably located in the IgE-binding region of Len c 3 by analogy with the Pru p 3 structure.

**Figure 5:**
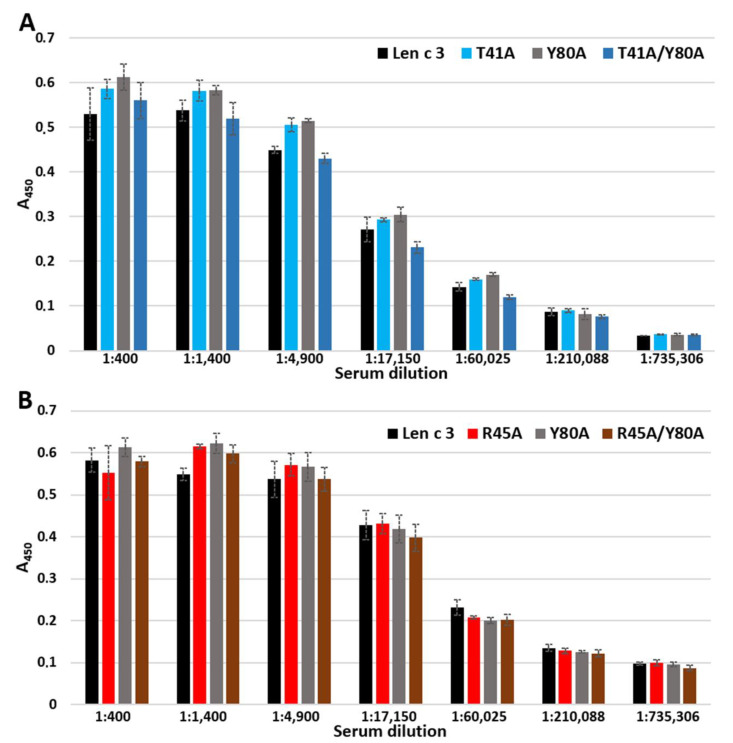
ELISA with Len c 3 and its mutant analogues using rabbit polyclonal anti-Len c 3 antibodies. (**A**) Comparison between Len c 3 and its mutant analogues T41A, Y80A and T41A/Y80A. (**B**) Comparison between Len c 3 and its mutant analogues R45A, Y80A and R45A/Y80A. Error bars represent standard deviation between technical replications.

Rabbit anti-Len c 3 antibodies [17] were used in ELISA assay in order to compare immunological properties of Len c 3 and its mutant analogues. It was shown that polyclonal rabbit anti-Len c 3 IgG bound to Len c 3 and all mutant analogues with equal effectiveness (Figure 5A,B). Thus, the replacement of one or two amino acid residues did not affect the interaction of mutant analogues with polyclonal rabbit anti-Len c 3 IgG.

**Figure 6:**
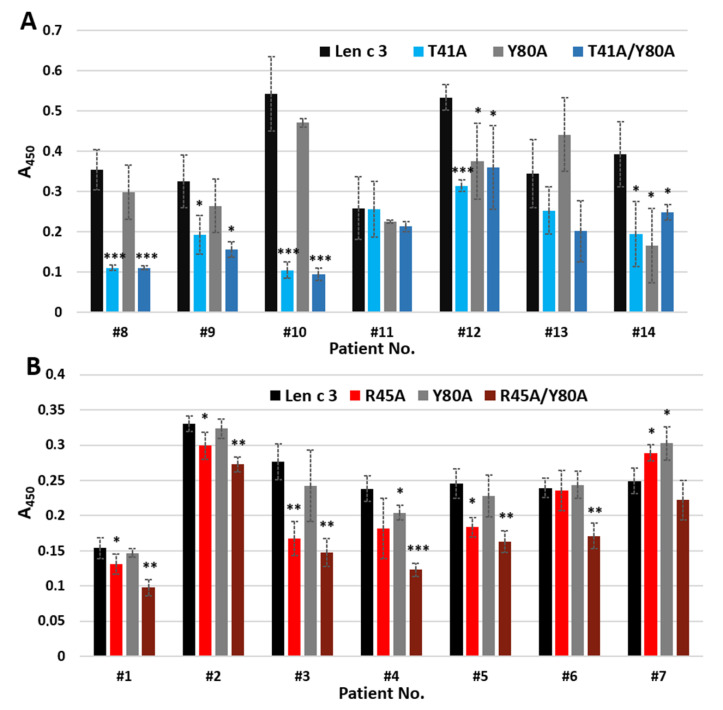
ELISA with Len c 3 and its mutant analogues using the patient sera containing sIgE to this allergen. (**A**) Comparison between Len c 3 and its mutant analogues T41A, Y80A and T41A/Y80A. (**B**) Comparison between Len c 3 and its mutant analogues R45A, Y80A and R45A/Y80A. Error bars represent standard deviation between technical replications. Differences in mean A values between Len c 3 and its mutant analogues for each patient were compared by *t*-test. Significance levels are: * *p* < 0.05; ** *p* < 0.01; *** *p* < 0.005.

Another result was obtained by ELISA IgE-binding assay performed with the use of sera from 14 allergic patients containing sIgE to Len c 3 (Figure 6). The Y80A analogue interacted with IgE with the same effectiveness as Len c 3 in most cases (Figure 6A,B). At the same time, a slight decrease of IgE-binding capacity was observed for the R45A analogue in four cases (Figure 6B). This effect was more pronounced for the mutant analogue T41A and for the double-mutant analogues R45A/Y80A and T41A/Y80A (Figure 6A).

## 4. Discussion

Lipid transfer proteins (LTPs) represent one of the most clinically significant classes of plant allergens. LTPs have a compact structure stabilized by four disulfide bonds, which provide them with a high stability. An important characteristic feature of these allergens is the presence of a hydrophobic cavity capable of accommodating different lipid ligands. The formation of complexes with ligands can affect the structure and allergenic properties of plant LTPs.

Earlier, it was shown that substitutions of such amino acids as Arg39, Thr40 and Arg44 in Pru p 3 structure led to substantial decrease (approximately 5 times) of IgE-binding capacity of this peach allergen [15]. It was also demonstrated that two protein regions 35–46 and 76–79 comprise major IgE-binding conformational epitope of Pru p 3 [24]. In the other publications, an important role of such amino acids as Asn35, Arg44 of Pru p 3 [25] and Arg46, Tyr81 of walnut allergen Jug r 3 and maize allergen Zea m 14 (walnut numbering) in the binding of lipid ligands was shown [26]. However, in our previous work [16], we assumed that the same amino acids in the LTP structure may play a key role both in the interaction with lipid ligands and specific IgE from sera of allergic patients.

In this work, on the example of lentil Len c 3, we investigated the effect of point amino acid substitutions simultaneously on the structural and allergenic properties of allergen of LTP class as well as on its ligand-binding capacity. For this aim in view, we obtained mutant analogues of the protein with single or double substitutions of three amino acid residues—Thr41, Arg45 and Tyr80 by site-directed mutagenesis. These amino acid residues were selected for several reasons. First, these residues are close to each other and located near the “bottom” entrance to the hydrophobic cavity of Len c 3 (Figure 3). Second, all of them are included in a presumable conformational epitope of Len c 3 predicted by analogy with Pru p 3 [15] (Figure 3). Third, Arg45 and Tyr80 play an important role in the interaction with lipids and stabilization of protein–ligand complexes in various LTPs [27], including Len c 3 [18]. Fourth, Tyr80 is one of the key residues for duodenal degradation of plant LTPs [28], including Len c 3 [13]. To meet this goal, we investigated structural properties of mutant analogues, their ability to bind lipid ligands and a rate of degradation under conditions mimicking digestion in the human gastrointestinal tract, as well as IgE-binding capacity.

Five mutant analogues of Len c 3 (T41A, R45A, Y80A, T41A/Y80A, R45A/Y80A) were obtained by site-directed mutagenesis and structurally characterized using CD-spectroscopy and computer modeling. The CD spectra revealed that all the mutant analogues did not have significant changes in secondary structures compared to the wild-type protein (Figure 1). Computer visualization of the mutant analogues demonstrated that some amino acid substitutions substantially affected the volume of the protein hydrophobic cavity. Substitution of Tyr80 (but not Thr41 and Arg45) by alanine led to an increase in the volume of the hydrophobic cavity. The Y80A, T41A/Y80A, and R45A/Y80A analogues had approximately the same sizes of hydrophobic cavities, which were extended compared to Len c 3. In addition, visualization showed that substitutions of selected amino acid residues affected the size and shape of the “bottom” entrance to the hydrophobic cavity. It was also noted that the replacement of Arg45, and to a greater extent of Thr41, but not Tyr80, led to the disruption of continuity of possible conformational IgE-binding epitope of Len c 3 (Figure 3). Based on these data, we hypothesized that substitutions of all the three amino acids could affect the ability of Len c 3 to bind lipid ligands. At the same time, Thr41 and Arg45 substitutions can alter an ability of the allergen to bind IgE from sera of allergenic patients. Verification of these assumptions was performed by comparative studies of lipid binding in vitro using fluorescence spectroscopy and immunological properties using enzyme-linked immunosorbent assay.

In vitro TNS displacement assay with fatty acids of different chain length and degree of saturation (PAL, OLE, and LIN) demonstrated that the replacement of both Arg45 and Tyr80 did not strongly affect an ability of the proteins to bind FAs. The mutant analogue with double substitution R45A/Y80A bound all these FAs less effectively than Len c 3 (Figure 2). The results obtained in this work are in good agreement with our previous data on the binding of lauric and stearic acids. An unexpected result was obtained with the mutant analogue T41A, which bound the tested FAs less effectively than Len c 3. Thr41 has not been previously described as an amino acid residue playing an important role in lipid binding. This effect was not observed for the T41A/Y80A analogue. A possible reason for the observed decrease or increase in the ability to bind fatty acids by mutant analogues may be confluence of the following factors: (1) a change in the size and shape of the hydrophobic cavity, which can lead both to spatially more favorable placement of the ligand in the hydrophobic cavity and to constriction in the interactions holding it inside; (2) a change in the size and shape of the “bottom” entrance to the cavity, which may facilitate or prevent the penetration of structurally different ligands into the cavity; (3) changes in the distribution of charges at the “bottom” entrance to the cavity, which play an important role in initiating interactions with ligands.

In ELISA experiments, changes in the ability of mutant analogues to bind polyclonal rabbit anti-Len c 3 IgG antibodies were not observed (Figure 5). However, when using sera from allergic patients containing specific IgE to Len c 3, we found that substitution of Arg45 and especially Thr41, but not Tyr80, by alanine resulted in a decrease in the IgE-binding capacity of mutant analogues (Figure 6). The IgE-reactivity of the T41A and T41A/Y80A analogues was similarly reduced. At the same time, the ability of R45A/Y80A to bind sIgE was reduced to a greater extent than in the case of the R45A analogue. We assumed that Thr41 plays the most important role in the interaction with sIgE among the three substituted amino acid residues apparently included in the conformational epitope of Len c 3. At the same time, amino acid substitutions leading to a significant, as in the case of the R45A/Y80A analogue, disruption of the structure of this epitope, can also crucially affect the IgE-binding capacity and the allergenic potential of this protein.

In the last part of the work, we investigated the effects of selected amino acid substitutions on the degradation rate of Len c 3 in experiments simulating its digestion in the human gastrointestinal tract in vitro. For the R45A and Y80A, the expected decrease in the rate of duodenal protein degradation was observed, since both Arg45 and Tyr80 form the cleavage recognition sites for such serine proteases as trypsin and α-chymotrypsin, respectively. At the same time, a cleavage rate of the R45A/Y80A analogue was not further reduced and was approximately the same as for the R45A and Y80A analogues. We hypothesized that a possible reason for this could be a significant change in the compactness of the structure of the R45A/Y80A analogue. This was in good agreement with data obtained by computer modeling of the largest cavity size for this mutant analogue. An unpredictable result was obtained for the T41A mutant analogue that was digested faster than wild-type protein. At the same time, cleavage of the double-mutant analogue T41A/Y80A was slower than that of Len c 3, but faster than cleavage of the Y80A analogue. We suggested that the substitution of Thr41 led to significant changes in the allergen conformation and an increase in the availability of cleavage sites for digesting enzymes. These results for Thr41 were in good agreement with the data on the decrease in the ability of the T41A analogue to bind lipids, as well as sIgE from sera of allergic patients.

## 5. Conclusions

In this study, using lentil Len c 3 and its mutant analogues (T41A, R45A, Y80A, T41A/Y80A and R45A/Y80A), we investigated the effects of substitutions of three amino acids—Thr41, Arg45 and Tyr80, spaced close to each other and located at the “bottom” entrance to the hydrophobic cavity of the protein, on its structural and allergenic properties as well as on its ligand-binding capacity. We showed that substitutions of three selected amino acids significantly affected the allergen structural organization and protein packing density, the size and shape of the hydrophobic cavity and the “bottom” entrance to it, as well as the structure of a possible conformational IgE-binding epitope. This, in turn, led to a change in the ability of Len c 3 to bind various ligands, in a rate of its proteolytic cleavage, and in the IgE-binding capacity of the allergen. Altogether, this led to a change in the allergenic potential of the protein. Taking into account all the data obtained, we conclude that site-directed mutagenesis of the region, participating in interactions of plant LTPs both with lipids and IgE, may in the future become a promising approach for modeling the structural and biological properties of allergens of this class and creating their hypoallergenic analogues.

## Data Availability

All data generated and analyzed during this study are included in this published article and its supplementary information file.

## Supplementary Materials

The following data are available online at https://www.mdpi.com/article/10.3390/membranes11120939/s1, Table S1: List of mutagenesis primers; Table S2. Secondary structure evaluation (%) predicted from Far-UV CD spectra; Figure S1: MALDI mass spectra of the Len c 3 and its mutant analogues.

## References

[1] Sicherer S.H., Sampson H.A. (2010). Food allergy. J. Allergy Clin. Immunol..

[2] Van Hengel A.J. (2007). Food allergen detection methods and the challenge to protect food-allergic consumers. Anal. Bioanal. Chem..

[3] Burks W., Ballmer-Weber B.K. (2006). Food allergy. Mol. Nutr. Food Res..

[4] van Esch B.C., Knipping K., Jeurink P., van der Heide S., Dubois A.E., Willemsen L.E., Garssen J., Knippels L.M. (2011). In vivo and in vitro evaluation of the residual allergenicity of partially hydrolysed infant formulas. Toxicol. Lett..

[5] Furrie E. (2005). Probiotics and allergy. Proc. Nutr. Soc..

[6] Kakleas K., Luyt D., Foley G., Noimark L. (2020). Is it necessary to avoid all legumes in legume allergy?. Pediatr. Allergy Immunol..

[7] Ireneo M.M.S., Ibáñez M.D., Sánchez J.-J., Carnés J., Fernández-Caldas E. (2008). Clinical features of legume allergy in children from a Mediterranean area. Ann. Allergy Asthma Immunol..

[8] Pascual C.Y., Fernandez-Crespo J., Sanchez-Pastor S., Padial M., Diaz-Pena J.M., Martin-Muñoz F., Martin-Esteban M. (1999). Allergy to lentils in Mediterranean pediatric patients. J. Allergy Clin. Immunol..

[9] Kalogeromitros D., Armenaka M., Galatas I., Capellou O., Katsarou A. (1996). Anaphylaxis Induced by Lentils. Ann. Allergy Asthma Immunol..

[10] Akkerdaas J., Finkina E., Balandin S., Magadán S.S., Knulst A., Fernandez-Rivas M., Asero R., Van Ree R., Ovchinnikova T. (2012). Lentil (*Lens culinaris*) Lipid Transfer Protein Len c 3: A Novel Legume Allergen. Int. Arch. Allergy Immunol..

[11] Gizatullina A.K., Finkina E.I., Mineev K.S., Melnikova D.N., Bogdanov I.V., Telezhinskaya I.N., Balandin S.V., Shenkarev Z.O., Arseniev A.S., Ovchinnikova T.V. (2013). Recombinant production and solution structure of lipid transfer protein from lentil Lens culinaris. Biochem. Biophys. Res. Commun..

[12] Melnikova D.N., Mineev K.S., Finkina E.I., Arseniev A.S., Ovchinnikova T.V. (2016). A novel lipid transfer protein from the dillAnethum graveolensL.: Isolation, structure, heterologous expression, and functional characteristics. J. Pept. Sci..

[13] Finkina E.I., Melnikova D.N., Bogdanov I.V., Matveevskaya N.S., Ignatova A.A., Toropygin I.Y., Ovchinnikova T.V. (2020). Impact of Different Lipid Ligands on the Stability and IgE-binding Capacity of the Lentil Allergen Len c 3. Biomol..

[14] Ge X., Chen J., Sun C., Cao K. (2003). Preliminary study on the structural basis of the antifungal activity of a rice lipid transfer protein. Protein Eng..

[15] García-Casado G. (2003). Identification of IgE-binding epitopes of the major peach allergen Pru p3. J. Allergy Clin. Immunol..

[16] Bogdanov I.V., Shenkarev Z.O., Finkina E.I., Melnikova D.N., Rumynskiy E.I., Arseniev A.S., Ovchinnikova T.V. (2016). A novel lipid transfer protein from the pea Pisum sativum: Isolation, recombinant expression, solution structure, antifungal activity, lipid binding, and allergenic properties. BMC Plant Biol..

[17] Bogdanov I.V., Finkina E.I., Balandin S.V., Melnikova D.N., Stukacheva E.A., Ovchinnikova T.V. (2015). Structural and Functional Characterization of Recombinant Isoforms of the Lentil Lipid Transfer Protein. Acta Nat..

[18] Melnikova D., Bogdanov I., Ignatova A., Ovchinnikova T., Finkina E. (2020). New insights into ligand binding by plant lipid transfer proteins: A case study of the lentil Lc-LTP2. Biochem. Biophys. Res. Commun..

[19] Laemmli U.K. (1970). Cleavage of Structural Proteins during the Assembly of the Head of Bacteriophage T4. Nature.

[20] DeLano W.L. (2002). Pymol: An open-source molecular graphics tool. CCP4 Newsl. Protein Crystallogr..

[21] Pravda L., Sehnal D., Toušek D., Navrátilová V., Bazgier V., Berka K., Vařeková R.S., Koča J., Otyepka M. (2018). MOLEonline: A web-based tool for analyzing channels, tunnels and pores (2018 update). Nucleic Acids Res..

[22] Eisenberg D., Schwarz E., Komaromy M., Wall R. (1984). Analysis of membrane and surface protein sequences with the hydrophobic moment plot. J. Mol. Biol..

[23] Gharibzahedi S.M.T., Mousavi S.M., Jafari S.M., Faraji K. (2012). Proximate composition, mineral content, and fatty acids profile of two varieties of lentil seeds cultivated in Iran. Chem. Nat. Compd..

[24] Pacios L.F., Tordesillas L., Cuesta-Herranz J., Compes E., Sánchez-Monge R., Palacín A., Salcedo G., Díaz-Perales A. (2008). Mimotope mapping as a complementary strategy to define allergen IgE-epitopes: Peach Pru p 3 allergen as a model. Mol. Immunol..

[25] Cuevas-Zuviría B., Garrido-Arandia M., Díaz-Perales A., Pacios L.F. (2019). Energy Landscapes of Ligand Motion Inside the Tunnel-Like Cavity of Lipid Transfer Proteins: The Case of the Pru p 3 Allergen. Int. J. Mol. Sci..

[26] Dubiela P., Del Conte R., Cantini F., Borowski T., Aina R., Radauer C., Bublin M., Hoffmann-Sommergruber K., Alessandri S. (2019). Impact of lipid binding on the tertiary structure and allergenic potential of Jug r 3, the non-specific lipid transfer protein from walnut. Sci. Rep..

[27] Melnikova D.N., Finkina E.I., Bogdanov I.V., Ovchinnikova T.V. (2018). Plant Pathogenesis-Related Proteins Binding Lipids and Other Hydrophobic Ligands. Russ. J. Bioorganic Chem..

[28] Abdullah S.U., Alexeev Y., Johnson P.E., Rigby N.M., Mackie A.R., Dhaliwal B., Mills E.N.C. (2016). Ligand binding to an Allergenic Lipid Transfer Protein Enhances Conformational Flexibility resulting in an Increase in Susceptibility to Gastroduodenal Proteolysis. Sci. Rep..

