# Supplementary material for: Effect of Point Mutations on Structural and Allergenic Properties of the Lentil Allergen Len c 3
Source: Membranes (Basel). 2021 Nov 27;11(12):939. doi: 10.3390/membranes11120939 (PMC8703665; doi:10.3390/membranes11120939)
Supplement: Supplementary file 1 [file membranes-11-00939-s001.zip › membranes-1466530-supplementary.pdf]

Supplementary Materials

Effect of Point Mutations on Structural and Allergenic Properties of the Lentil Allergen Len c 3

Daria N. Melnikova [1,2,*], Ekaterina I. Finkina [1], Ivan V. Bogdanov [1], Anastasia A. Ignatova [1], Natalia S. Matveevskaya [3], Andrey A. Tagaev [1] and Tatiana V. Ovchinnikova [1,2]

[1] M.M. Shemyakin & Yu.A. Ovchinnikov Institute of Bioorganic Chemistry, The Russian Academy of Sciences, Miklukho-Maklaya Str., 16/10, 117997 Moscow, Russia; finkina@mail.ru (E.I.F.); contraton@mail.ru (I.V.B.); aignatova_83@mail.ru (A.A.I.); andazitag@yandex.ru (A.A.T.); ovch@ibch.ru (T.V.O.)
[2] Phystech School of Biological and Medical Physics, Moscow Institute of Physics and Technology (State University), 141701 Dolgoprudny, Russia
[3] G.N. Gabrichevsky Research Institute for Epidemiology and Microbiology, Admiral Makarov St., 10, 125212 Moscow, Russia; matveevskaya@mail.ru
* Correspondence: d_n_m_@mail.ru; Tel.: +7-495-335-42-00

Table S1. List of mutagenizing primers.

Mutation	Mutagenizing primer sequences (5′-3′)
sT41A	ACGCCTGATCGTCAGGCTGCCTG
ansT41A	GCAGCCTGACGATCAGGCGTTGCGTTGGCGGCAGCAAGAAGC T
sY80A	AAGATCAGTACCACCACCAACTG
ansY80A	TTGGTGGTGGTACTGATCTTTGCAGGAATGTTGACACCACATT

Table S2. Secondary structure evaluation (%) predicted from Far-UV CD spectra.

Sample	α-helix, %	β-sheet, %	β-turn, %	random, %	NRMSD*
Len c 3	28.4	19.1	22.1	30.3	0.01
T41A	29.3	18.1	22.3	30.1	0.02
R45A	29.2	18.6	21.8	30.5	0.01
Y80A	29.1	19.1	21.5	30.3	0.02
T41A/ Y80A	29.2	18.4	22.2	30.2	0.02
R45A/ Y80A	29.0	18.2	21.1	31.7	0.01

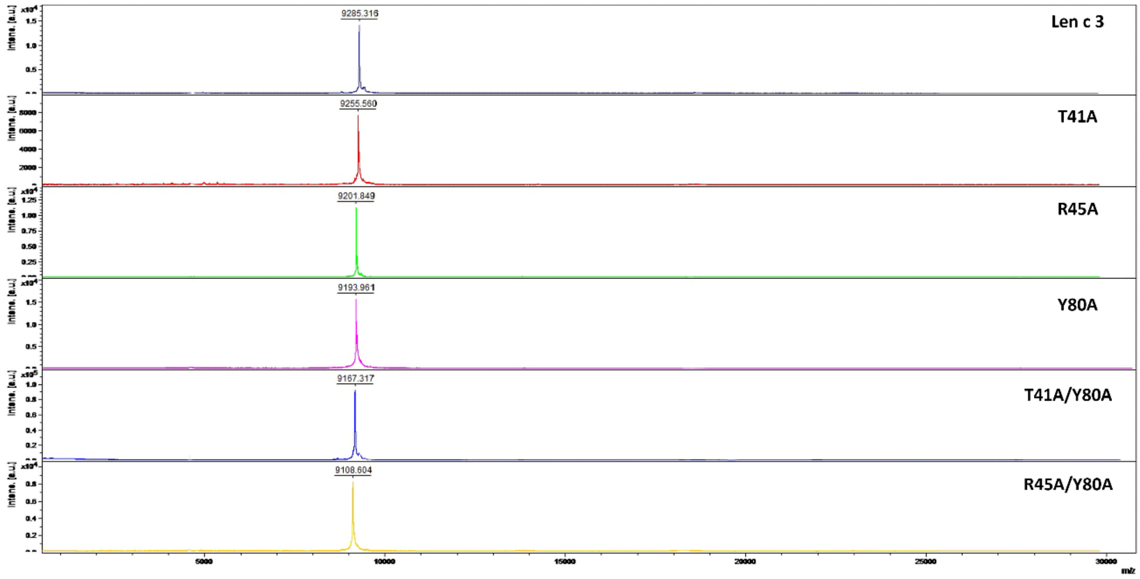

Figure S1. MALDI mass spectra of the Len c 3 and its mutant analogues.
